# Performance Assessment of Treponemal and Nontreponemal Tests for the Diagnosis of Acquired Syphilis

**DOI:** 10.4269/ajtmh.23-0238

**Published:** 2024-04-09

**Authors:** Ângelo Antônio Oliveira Silva, Ayla Araújo Lima, Larissa de Carvalho Medrado Vasconcelos, Rosângela Andrade de Almeida, Natália Erdens Maron de Freitas, Fernanda Lopes Habib, Talita Andrade Oliva, Miralba Freire de Carvalho Ribeiro da Silva, Isadora Cristina de Siqueira, Fred Luciano Neves Santos

**Affiliations:** ^1^ Advanced Public Health Laboratory, Institute Gonçalo Moniz, Foundation Oswaldo Cruz (FIOCRUZ-BA), Salvador, Brazil;; ^2^ Salvador University (UNIFACS), Salvador, Brazil;; ^3^ State Center Specializing in Diagnosis, Assistance, and Research (CEDAP), Salvador, Brazil;; ^4^ Laboratory of Experimental Pathology, Institute Gonçalo Moniz, Foundation Oswaldo Cruz (FIOCRUZ-BA), Salvador, Brazil;; ^5^ Integrated Translational Program in Chagas Disease from Fiocruz (Fio-Chagas), Rio de Janeiro, Brazil

## Abstract

There are a variety of nontreponemal test (NTT) and treponemal test (TT) kits for the serologic diagnosis of syphilis. Because of the complexity of the infection (multiple clinical stages) and the different antigens used in these kits, a systematic evaluation of the accuracy of the currently available commercial tests is warranted. Our objective was to evaluate the performance of commercially available tests for the diagnosis of syphilis infection. In this study, we analyzed one NTT (Venereal Disease Research Laboratory [VDRL] test, Wiener Laboratories, Rosario, Argentina) and two TTs (fluorescent treponemal antibody absorption [FTA-ABS] test, Euroimmun, Lübeck, Germany, and syphilis recombinant ELISA v. 4.0 test [ELISA], Wiener Laboratories, Rosario, Argentina) using a panel of 187 samples, including serum samples from 31 individuals with primary syphilis, 77 with secondary syphilis, and 79 with latent syphilis. An additional 192 samples from uninfected individuals and 323 serum samples from individuals with other diseases were included. The sensitivities of the VDRL, ELISA, and FTA-ABS tests were 97.9%, 100%, and 96.3%, respectively. The VDRL and ELISA tests showed a specificity of 100%, and the FTA-ABS test showed a specificity of 99.5%. Accuracy was 98.9% for the VDRL test, 100% for the ELISA, and 97.9% for the FTA-ABS test. For primary, secondary, and latent syphilis, the ELISA achieved a diagnostic performance of 100%, whereas the sensitivity for the VDRL and FTA-ABS tests ranged from 96.8% to 98.7% and 93.7% to 98.7%, respectively. No difference was observed when the tests were used as traditional or reverse algorithms. In general, all three tests are able to discriminate positive and negative samples for syphilis, regardless of the diagnostic algorithm.

## INTRODUCTION

Syphilis is a chronic bacterial infection caused by the spirochete *Treponema pallidum* subspecies *pallidum* (*T. pallidum*). It can be transmitted sexually by direct contact with syphilitic mucosal lesions (e.g., chancroid and condyloma lata) or via vertical transmission from mother to fetus during pregnancy. The disease represents a massive public health problem worldwide, with approximately 6.3 million new cases per year among women and men aged 15–49 years and an estimated global prevalence of 0.5% from 2012 to 2016.^1^ It is also the leading cause of childhood morbidity and mortality.^2^^,^^3^ In 2016, the estimated global prevalence of syphilis was 473 cases per 100,000 live births.^4^ The infection is divided into different stages according to clinical findings: primary, secondary, latent, and tertiary syphilis.^5^^–^^7^

*Treponema pallidum* cannot be cultured, and Food and Drug Administration-approved PCR is not available for *T. pallidum* and is not sensitive enough. Therefore, diagnosis is performed by serologies or dark-field microscopy.^8^ Dark-field examination of lesion exudate or tissue in the early stages of syphilis may lead to or support a diagnosis. However, because of its complexity, it is rarely performed today. Instead, serology is usually used. Serology is divided into two different tests: nontreponemal tests (NTTs) and treponemal tests (TTs). A single serologic NTT or TT is not sufficient for diagnosis. The reason for this is the high rate of false-positive results among persons without syphilis or with previously treated syphilis and false-negative results among persons tested during primary syphilis.^8^ Therefore, a correct diagnosis requires a combination of diagnostic tests and careful clinical assessment.^9^

Nontreponemal tests such as the Venereal Disease Research Laboratory (VDRL) test and the rapid plasma reagin (RPR) test are based on anticardiolipin antibody detection and are helpful in monitoring the stage of infection and treatment. They are easy to perform, inexpensive, and provide a rapid diagnostic result.^10^ However, they have low specificity because of cross-reactivity with antibodies produced in various autoimmune diseases or during pregnancy. Additionally, NTTs may not reliably detect early primary syphilis because of their lower sensitivity.^11^ On the other hand, TTSs such as the fluorescent treponemal antibody absorption (FTA-ABS) test, the ELISA, and the chemiluminescent magnetic microparticle immunoassay (CMIA) detect anti-*T. pallidum* IgG or IgM and are used for screening or as confirmatory tests to exclude false-positive results in NTTs. Moreover, they prove valuable in detecting early syphilis cases that NTTs may overlook.^10^^,^^12^ In comparison with NTTs, they are costly, cumbersome, automated, require a trained team to perform, and are not recommended for monitoring treatment progress, relapse, or reinfection in individuals previously treated, as they remain reactive for years, regardless of treatment.^11^^,^^13^^,^^14^

In light of this scenario, we endeavored to perform a systematic evaluation of the performance of commercial kits for diagnosing acquired syphilis. Statistical tools were used to obtain a robust assessment of the performance of each serological test by determining the following diagnostic test parameters: sensitivity (the probability that a test is positive in the presence of infection) and specificity (the probability that a test is negative in the absence of infection).

## MATERIALS AND METHODS

### Study population.

The sample size was calculated with a 95% CI, an expected sensitivity and specificity of 99%, and an absolute error of 1.5%. Based on these parameters, which were determined using the OpenEpi program (www.openepi.com),^15^ the minimum sample size required to conduct this study was 169 anti-*T. pallidum*-positive samples and 169 anti-*T. pallidum*-negative samples. The positive samples were from 187 patients diagnosed with syphilis between April 2021 and November 2021 at the State Center Specializing in Diagnosis, Assistance, and Research (CEDAP) in the state of Bahia, Brazil. The selection of patients was based on the positivity of serological tests (rapid immunochromatographic test and VDRL test) and confirmed clinical examination for syphilis performed at the reference center by physicians and nurses. According to their clinical status, patients were classified as follows: primary syphilis (*n =* 31), secondary syphilis (*n =* 77), and recent latent syphilis (*n =* 79). The anti-*T. pallidum*-negative samples were obtained from healthy blood donors (*n =* 192) at the Bahia State Blood Bank (HEMOBA) from December 2017 to April 2019. In addition, to evaluate cross-reactivity, 323 serum samples were also obtained from individuals with unrelated diseases, previously defined by serological diagnosis, from the serum banks of HEMOBA and the State Blood Bank of Pernambuco (HEMOPE), as follows: Chagas disease (*n =* 67), hepatitis B virus (HBV) (*n =* 91), hepatitis C virus (HCV) (*n =* 68), HIV (*n =* 24), and human T-cell lymphotropic virus (HTLV) (*n =* 68). Selection of samples for cross-reactivity evaluation was based on positivity for the disease analyzed and negativity for syphilis in the chemiluminescence assay used at the blood bank. Serum samples were stored in sealed, well-labeled microtubes at –20°C until immunoassays.

### Laboratory evaluation.

All samples used in the present study were tested with two treponemal tests (the FTA-ABS test used was the Anti-*Treponema pallidum* indirect immunofluorescence test [IIFT] IgG from Euroimmun Medizinische Labordiagnostika AG, Lübeck, Germany; the ELISA used was the Sífilis ELISA recombinante v. 4.0 test from Wiener Laboratories, Rosario, Argentina) and a nontreponemal test (the VDRL test from Wiener Laboratories, Rosario, Argentina). These kits were selected based on their commercial availability in the Brazilian market and their availability in stock at the time of purchase from the supplier. All three commercial tests were performed in strict accordance with the manufacturers’ specifications. Briefly, for the VDRL assay, 50-*μ*L volumes of the samples or controls were mixed with one drop of the aqueous buffered suspension of purified cardiolipin antigen and lecithin in a well of the excavated slide. The plate was then shaken horizontally at 180 rpm for 4 minutes and immediately observed under the microscope at ×60 to ×100 magnification. For the semiquantitative assay, serial dilutions of the samples were performed in 0.9% sodium chloride. For the FTA-ABS test, 12.5-*μ*L volumes of the samples were added to plastic microtubes along with 50 *μ*L of the FTA sorbent. The tubes were vortexed for 4 seconds and incubated at 37°C in a water bath for 30 minutes. After incubation, 25 *μ*L of the supernatant was transferred to another microtube and 25 *μ*L of phosphate-buffered saline plus Tween (PBS-Tween) was added. The tubes were vortexed again for 4 seconds. Then, 30 *μ*L of the diluted sample was pipetted into each well of the sensitized slide, which was incubated in a humidity chamber at room temperature for 30 minutes. The slides were washed and immersed in a cuvette containing PBS-Tween for 5 minutes. After washing, 25 *μ*L of a fluorescein-conjugated antibody was added to each well and the slides were again incubated in a humidity chamber for 30 minutes at room temperature. The slides were washed, dried, and prepared for mounting by adding one drop of glycerol to each reaction well and then placing a coverslip on top. Measurements were performed using a fluorescence microscope with 20× and 40× objectives and an excitation filter between 450 and 490 nm. For the recombinant ELISA, serum samples and controls (120 *μ*L) were added to the sample dilution buffer at a ratio of 1:6 and placed in the polycuvettes, followed by incubation at 37°C for 60 minutes. The cuvettes were then washed with wash buffer, and 100 *μ*L of the developer was added, followed by incubation for 30 minutes at room temperature in the dark. The stop solution was then added to the polycuvettes, and absorbance values were measured in a spectrophotometer at 450 nm. Cutoff values were determined for each plate using the following calculation: cutoff value = NC (negative control) + 0.160.

### Screening algorithms.

Considering the TTs and NTTs analyzed in this study, anti-*T. pallidum*-positive and -negative samples were evaluated according to two diagnostic algorithms: the traditional and the reverse. In the traditional algorithm, the VDRL test was used in the first level of testing in a screening algorithm. A negative VDRL test result excludes the disease, whereas positive results should be forwarded for diagnostic confirmation using the FTA-ABS test to exclude or confirm infection. Samples for which the dual-serology test in the traditional algorithm did not yield concordant results were tested with the ELISA. In the reverse algorithm, the FTA-ABS test was used in the first level of testing in a screening algorithm. A negative FTA-ABS result excludes the disease, whereas positive results should be forwarded for diagnostic confirmation using the VDRL test. Similar to the traditional algorithm, the ELISA was also used in the reverse algorithm for discordant results.

## STATISTICAL ANALYSES

**Figure 1.**
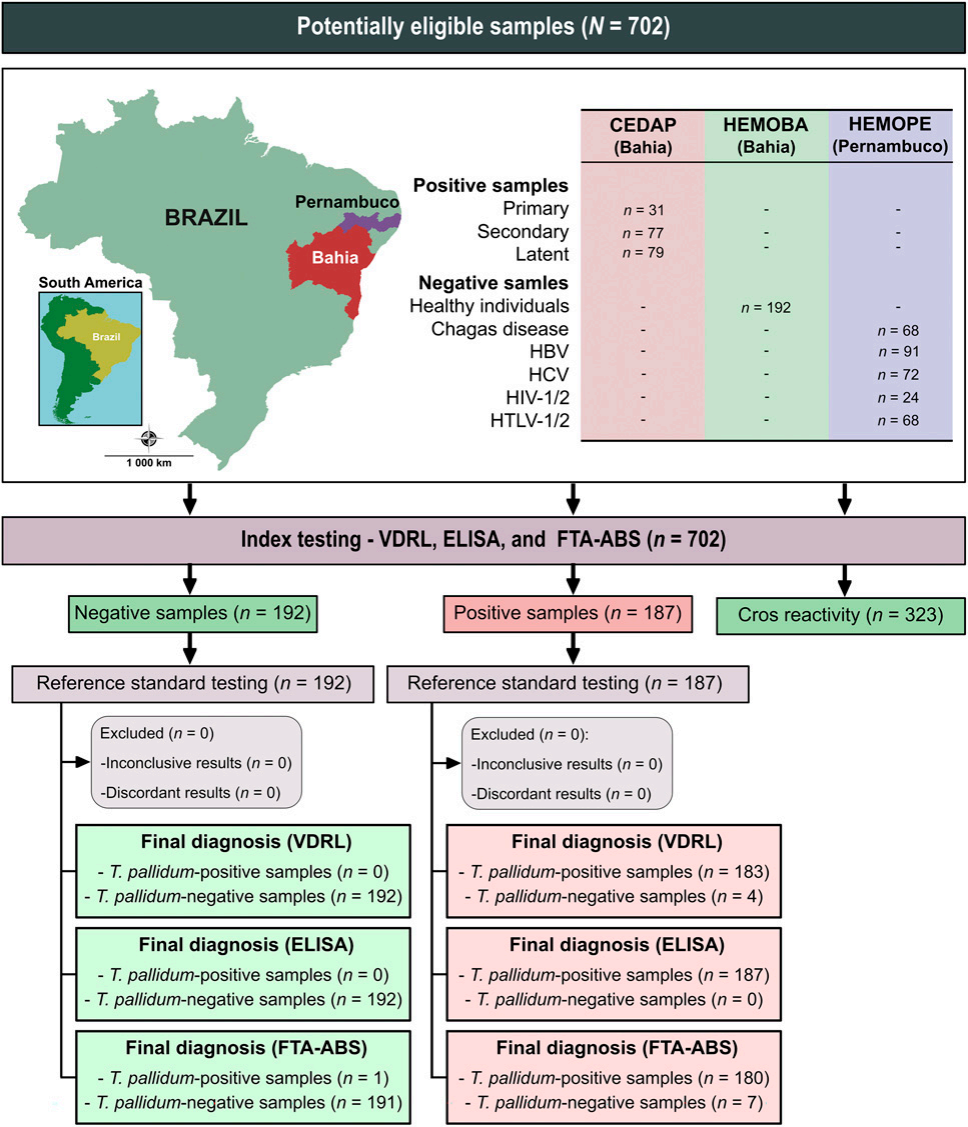
Flow chart illustrating study design in conformity with the Standards for Reporting of Diagnostic Accuracy Studies (STARD) guidelines. Source base layer and credit base layer: https://data.humdata.org/ published under Creative Commons attribution for intergovernmental organizations: https://data.humdata.org/dataset/geoboundaries-admin-boundaries-for-brazil.

The diagnostic performances of the tests (VDRL, ELISA, and FTA-ABS) were calculated using a dichotomous approach (2 × 2 contingency table) and compared in terms of sensitivity, specificity, accuracy, likelihood ratio, and diagnostic odds ratio (DOR). The assessment of imprecision was based on Cohen’s kappa coefficient (κ), which was interpreted as poor (κ ≤ 0.00), slight (0.00 < κ ≤ 0.20), fair (0.21 < κ ≤ 0.40), moderate (0.41 < κ ≤ 0.60), substantial (0.61 < κ ≤ 0.80), and almost perfect (0.81 < κ ≤ 1.0) agreement.^16^ The CI was used to account for the precision of the proportion estimates, and the CI was set at 95%. The screening algorithms were also evaluated based on the test agreement. Data were coded and analyzed using computer graphics software (GraphPad Prism v. 9; GraphPad, San Diego, CA). A flow chart (Figure 1) was prepared according to the Standards for Reporting of Diagnostic Accuracy Studies guidelines.^17^

## RESULTS

A total of 702 serum samples were tested with all three commercial IgG *T. pallidum* immunoassays: two treponemal tests (FTA-ABS IgG [Euroimmun] and recombinant ELISA v.4.0 [Wiener Laboratories) and one nontreponemal test (VDRL test [Wiener Laboratories]) (individual data points and results are available in Supplemental Table 1). Regardless of the clinical form of syphilis, anti-*T. pallidum* antibodies were detected in 96.3% (180/187) of anti-*T. pallidum*-positive individuals by the FTA-ABS test, in 97.9% (183/187) by the VDRL test, and in 100% (187/187) by the recombinant ELISA (Table 1). In anti-*T. pallidum*-negative samples, the highest value of specificity (100%) was obtained with the VDRL test and the the ELISA. The FTA-ABS test showed lower specificity than the other assays, but this difference was insignificant (99.5%). Accuracy reached the highest value when samples were tested with the ELISA (100%), the VDRL test (98.9%), and the FTA-ABS test (97.9%). Test performance was summarized by the DOR value, which reached 3,594,191 for the ELISA and 876,303 for the VDRL test. The FTA-ABS test achieved a DOR of 4,911, a significantly lower value than those of the other kits. Qualitative evaluation of the results using Cohen’s kappa method showed almost perfect agreement between all three kits and the reference tests. Considering the 95% CI overlap, the sensitivity, specificity, accuracy, and Cohen’s kappa index showed no differences between the three kits (Table 1).

**Table 1:** Diagnostic performance of three commercial IgG *T. pallidum* immunoassays for the diagnosis of syphilis by using anti-*T. pallidum*-positive (all clinical stages; *n =* 187) and anti-*T. pallidum*-negative (*n =* 192) samples

Performance Parameters	VDRL*	ELISA†	FTA-ABS‡
SEN % (95% CI)	97.9 (94.6 –99.2)	100 (98.0–100)	96.3 (92.5–98.2)
SPE % (95% CI)	100 (98.0–100)	100 (98.0–100)	99.5 (97.1–99.9)
ACC % (95% CI)	98.9 (97.3–99.6)	100 (99.0–100)	97.9 (95.9–98.9)
PLR	18,790	19,201	183
NLR	0.02	<0.01	0.04
DOR	876,303	359,077,901	4,956
K (95% CI)	0.98 (0.96–1.00)	1.00	0.96 (0.93–0.99)

ACC = accuracy; DOR = diagnostic odds ratio; FTA-ABS = fluorescent treponemal antibody absorption test; K = Cohen’s kappa coefficient; NLR = negative likelihood ratio; PLR = positive likelihood ratio; SEN = sensitivity; SPE = specificity; VDRL = Venereal Disease Research Laboratory test.

* VDRL test; Wiener Laboratories, Rosario, Argentina.

† Sífilis ELISA recombinante v. 4.0 test; Wiener Laboratories, Rosario, Argentina.

‡ Anti-*Treponema pallidum* IIFT IgG; Euroimmun Medizinische Labordiagnostika AG, Lübeck, Germany.

To evaluate the heterogeneity of IgG detection by all three commercial *T. pallidum* immunoassays due to different clinical stages of syphilis, sensitivities were compared using samples from individuals with primary, secondary, and early latent syphilis (Table 2). We found positivity in all 31 positive samples from individuals with primary syphilis tested with the recombinant ELISA, whereas 96.8% (30/31) of the samples tested with the VDRL and FTA-ABS tests were positive. Among samples from individuals with secondary syphilis (*n =* 77), ELISA detected IgG in 100% of samples, and both the VDRL and FTA-ABS tests detected IgG in 98.7% (76/77) of samples. Sensitivity in individuals with late latent disease (*n =* 79) was 100% for samples tested with the ELISA, 97.5% (77/79) for samples tested with the VDRL test, and 93.7% (74/79) for samples tested with the FTA-ABS test. Regardless of clinical stage, no significant differences were found between tests.

**Table 2:** Sensitivity of three commercial IgG *T. pallidum* immunoassays for the diagnosis of syphilis, by clinical stage

Anti-*T. pallidum*-Positive Samples by Clinical Stage	Sensitivity % (95% CI)		
	VDRL*	ELISA†	FTA-ABS‡
Primary syphilis (*n =* 31)	96.8 (83.8–99.4)	100 (89.0–100)	96.8 (83.8–99.4)
Secondary syphilis (*n =* 77)	98.7 (93.0–99.8)	100 (95.2–100)	98.7 (93.0–99.8)
Early latent syphilis (*n =* 79)	97.5 (91.2–99.3)	100 (95.4–100)	93.7 (86.0–97.3)

FTA-ABS = fluorescent treponemal antibody absorption test; VDRL = Venereal Disease Research Laboratory test.

* VDRL test; Wiener Laboratories, Rosario, Argentina.

† Syphilis recombinant ELISA v. 4.0 test; Wiener Laboratories, Rosario, Argentina.

‡ Anti-*Treponema pallidum* IIFT IgG; Euroimmun Medizinische Labordiagnostika AG, Lübeck, Germany.

All three commercial assays were performed to evaluate cross-reactivity against antibodies from unrelated diseases by using a panel of 323 serum samples (individual data points and results are available in Supplemental Table 2). As shown in Table 3, a higher incidence of cross-reactivity was observed when serum samples were tested with the ELISA and the FTA-ABS test, especially for sexually transmitted viral infections. The highest incidence in samples tested with the FTA-ABS test was observed for HCV (14.7%), followed by HIV (12.5%), HBV (8.8%), and HTLV (7.4%). No cross-reactivity was observed when the VDRL test was used.

**Table 3:** Cross-reactivity analysis of three commercial IgG *T. pallidum* immunoassays with serum samples from individuals affected by unrelated infections

Samples (*n*)	VDRL*	ELISA†	FTA-ABS‡
Other infections (*n =* 323)	0.0%	2.8% (9/323)	8.0% (26/323)
Chagas disease (*n =* 68)	0.0%	2.9% (2/68)	0.0%
HBV (*n =* 91)	0.0%	3.3% (3/91)	8.8% (8/91)
HCV (*n =* 68)	0.0%	2.9% (2/68)	14.7% (10/68)
HIV (*n =* 24)	0.0%	0.0%	12.5% (3/24)
HTLV (*n =* 68)	0.0%	2.9% (2/68)	7.4% (5/68)

FTA-ABS = fluorescent treponemal antibody absorption test; HBV = hepatitis B virus; HCV = hepatitis C virus; HTLV = human T-cell lymphotropic virus; VDRL = Venereal Disease Research Laboratory test.

**Figure 2.**
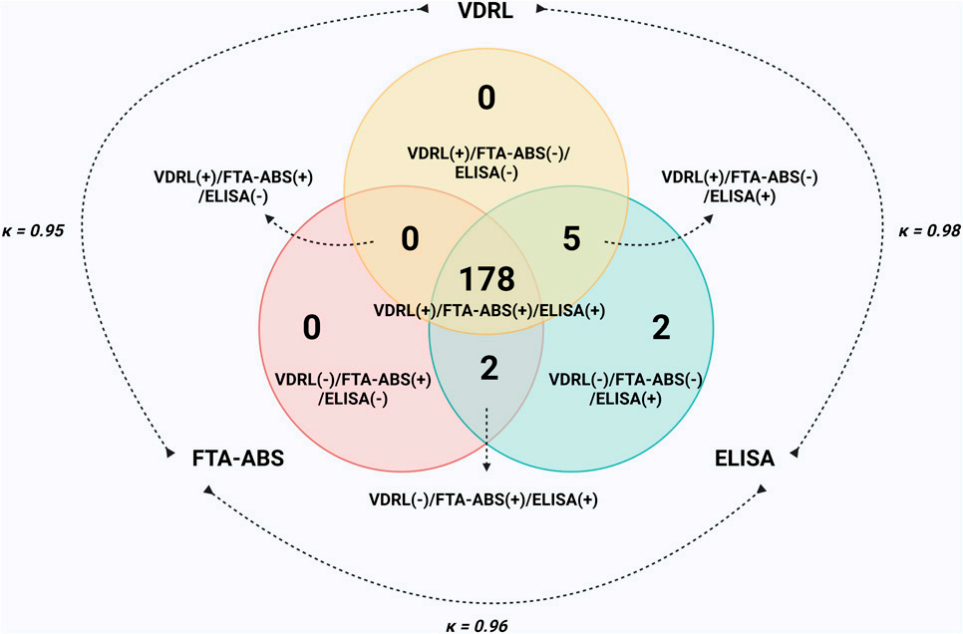
Venn diagram evaluating the imprecision of three commercial IgG *T. pallidum* immunoassays for the diagnosis of syphilis using anti-*T. pallidum*-positive samples (all clinical stages; *n =* 187).

The assessment of imprecision between all three commercial tests is shown in a Venn diagram in Figure 2. The highest Cohen’s kappa index (κ) was obtained for ELISA + VDRL [κ = 0.98 (95% CI, 0.96–1.00)], followed by ELISA + FTA-ABS [κ = 0.96 (95% CI, 0.93–0.99)] and FTA-ABS + VDRL [κ = 0.95 (95% CI, 0.91–0.98)]. In general, all tests showed high diagnostic performance in serologic recharacterization and discriminated well between positive and negative samples for syphilis.

**Figure 3.**
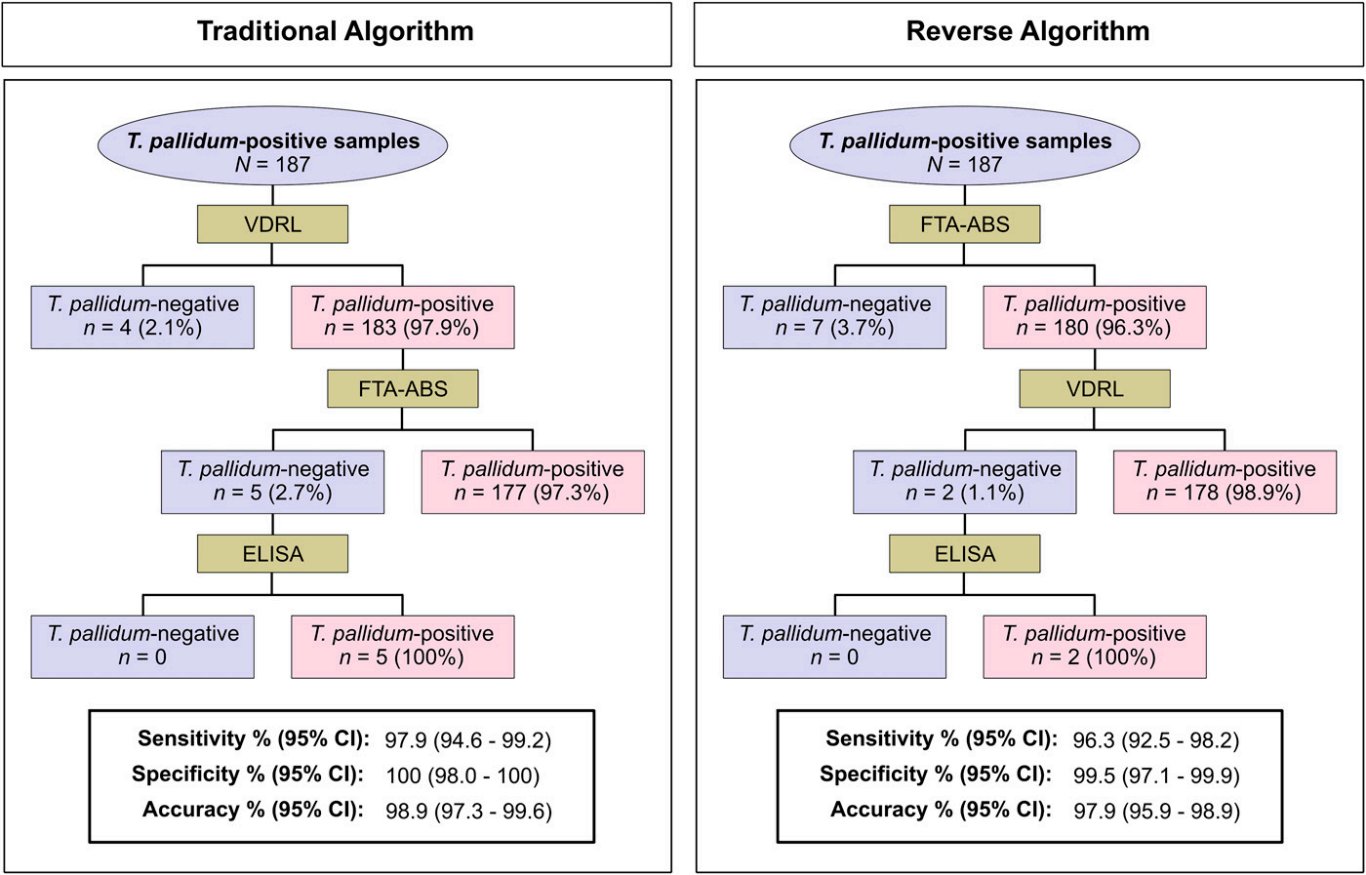
Comparison of performance parameters of the traditional and reverse algorithms considering anti-*T. pallidum*-positive (all clinical stages; *n =* 187) and anti-*T. pallidum*-negative (*n =* 192) samples. All anti-*T. pallidum*-negative samples tested negative in the traditional algorithm using the Venereal Disease Research Laboratory (VDRL) test, whereas one sample tested false-positive in the reverse algorithm using the fluorescent treponemal antibody absorption (FTA-ABS) test.

The performances of the traditional and reverse algorithms were also evaluated. In the traditional algorithm, the VDRL test result excluded the disease in four (2.1%) of 187 anti-*T. pallidum*-positive samples. Subsequently, five (2.7%) and 177 (97.3%) samples tested negative and positive, respectively, using the FTA-ABS test. All five samples that tested negative with the FTA-ABS test proved positive when tested with the ELISA. Using the reverse algorithm, the FTA-ABS test diagnosed seven (3.7%) anti-*T. pallidum*-positive samples as negative. The other 180 correctly diagnosed anti-*T. pallidum*-positive samples were tested with the VDRL test, which confirmed positivity in 178 (98.9%) samples. Two anti-*T. pallidum*-positive samples that tested negative by the VDRL test proved positive by the ELISA. Diagnostic parameters were compared regarding sensitivity, specificity, and accuracy, but no differences were found between the two screening algorithms (Figure 3). Almost perfect agreement was found between the traditional and reverse algorithms (κ = 0.97 [95% CI, 0.94–0.99]).

## DISCUSSION

Although numerous treponemal and nontreponemal assays are currently commercially available, a systematic evaluation of the performance of commercial kits for the diagnosis of acquired syphilis is required, because few studies of immunoassays have included clinically characterized samples stratified by stage. Therefore, in the present study, we evaluated the diagnostic performance of a nontreponemal assay (VDRL test) and two treponemal assays (ELISA and FTA-ABS test) to detect antibodies in the sera of anti-*T. pallidum*-positive and -negative individuals.

Performance evaluations were performed using the VDRL test to determine diagnostic sensitivity, specificity, and accuracy for syphilis. Considering all clinical stages of syphilis, the VDRL test showed high sensitivity, specificity, and accuracy values. In fact, only four anti-*T. pallidum*-positive samples were falsely classified as negative. Compared with the VDRL test, the treponemal tests evaluated also showed false-negative results. Of 187 tested samples, only seven were classified as negative by the FTA-ABS test, whereas all were correctly classified as positive by the ELISA, with no false-negative results. As with the VDRL test, similarly high sensitivity values (100%) were found in other studies,^18^^,^^19^ whereas specificity ranged from 64.1% to 100%.^18^^–^^20^ In contrast, sensitivity (49.4% and 77.1%)^20^^–^^22^ and accuracy (71.1%) were lower in other studies.^23^ Here, we found high diagnostic sensitivity (>96%) for all stages when samples were stratified by clinical stage. These results are not consistent with the results of other studies. For example, a 2006 study described low sensitivity for primary syphilis (70%) and syphilis of unknown duration (52%), except for secondary syphilis (96%) and early latent syphilis (87%).^22^ Another study showed varying sensitivity values from 62.5% to 78.4% for primary syphilis and from 85% to 100% for early latent syphilis but a high value (100%) for secondary syphilis.^24^ The VRDL test was more sensitive than other NTTs such as the RPR. Different sensitivity and specificity values were described for the RPR, ranging from 57.7% to 100%^25^^–^^28^ and from 57.1% to 96.19%,^26^^,^^27^^,^^29^ respectively. These differences may be attributed to the number of samples analyzed, which may impact the statistical results presented, to unknown clinical and epidemiologic histories, because early primary syphilis can be missed due to the lower sensitivity of NTTs,^11^ to cross-reactivity that decreases the specificity due to antibodies produced in various autoimmune diseases or during pregnancy,^11^ to the prozone phenomenon, which causes false-negative results, and to the use of the stained RPR (technical limitation). The samples used in the present study were well characterized, thus excluding the potential biases described above.

We also evaluated the performance of two TTs (ELISA and FTA-ABS). For the ELISA, high sensitivity, specificity, and accuracy values were obtained (100%). Other studies also showed similar performance values. Using different ELISA brands (Trep-Check IgG enzyme immunoassay [EIA] Trep-Sure, ICE Syphilis detection pack, Trep-ID, TS-EIA, Cobas Syphilis EIA, TP-ELISA, Enzywell TP, Murex ICE EIA, Vircell Syphilis ELISA IgG+IgM, DIALAB ELISA, RecomWell IgG, Architect Syphilis Tp ELISA, Murex ICE, Chorus Syphilis Screen Recombinant, Advia Centaur syphilis assay, and Euroimmun *Treponema pallidum* Screen ELISA), sensitivity was found to range from 83% to 100%, specificity ranged from 80% to 100%,^20^^,^^22^^,^^26^^,^^27^^,^^29^^–^^39^ and accuracy was 95.4%.^26^ However, other studies showed lower sensitivity for the TS-EIA (52%)^25^ and the Vircell Syphilis ELISA IgG+IgM (73.2%)^30^ and for the specificity of the ICE Syphilis detection pack (61.5%), Enzywell TP (59%),^32^ and Vircell Syphilis ELISA IgG+IgM (62.6%).^34^ Here, ELISA v. 4.0 was 100% accurate in diagnosing all clinical stages. However, Murex ICE EIA showed 84%, 100%, 75%, and 100% sensitivity for primary syphilis, secondary syphilis, early latent syphilis, and syphilis of unknown duration, respectively.^22^ In another study, similar values (100%) were obtained for secondary and early latent syphilis, whereas sensitivity for primary syphilis was lower (80%).^40^ For Trep-Sure, recomWell, Syphilis Screening ELISA, and Enzywell, values above 94% were observed at all stages.^21^^,^^39^^,^^41^

As with the ELISA, the FTA-ABS test showed high performance. However, seven anti-*T. pallidum*-positive samples from the FTA-ABS test were falsely diagnosed as negative, with a sensitivity of 96.3%. These seven false-negative samples were correctly classified as positive by the ELISA, and five of them were classified as positive by the VDRL test. Of the anti-*T. pallidum*-negative samples, only one was positive when assessed by the FTA-ABS test, with a specificity of 99.5%. Our findings are consistent with those of other studies that reported sensitivity values ranging from 86.1% to 100%,^19^^,^^21^^,^^29^^,^^30^^,^^32^^,^^34^^,^^37^^,^^39^ and specificity ranged from 92.5% to 100%.^18^^,^^19^^,^^29^^,^^30^^,^^34^^,^^37^^,^^39^ In contrast, our results are not consistent with data from other studies, which reported sensitivity and specificity values of 77.5%^18^ and 61.5%,^32^ respectively. When the serum samples were stratified by clinical stage of syphilis, we found that sensitivity ranged from 93.7% for latent syphilis to 96.8% and 98.7% for primary and secondary syphilis. Other studies reached similar results, except for those for primary syphilis, which had a low value (∼78%).^39^^,^^41^

Considering Cohen’s kappa index (κ), the highest degree of agreement was observed between the recombinant ELISA and the VDRL test (κ = 0.98), followed by the recombinant ELISA and the FTA-ABS test (κ = 0.96) and by the FTA-ABS and VDRL tests (κ = 0.95). Indeed, almost perfect agreement (κ = 0.84) was also observed between other TTs (CMIA) and the VDRL test^18^ and between the ELISA, the RPR, and the RDT (Rapid diagnostic test) (κ = 0.93).^38^ Similar results (κ = 0.90–0.96) were also observed for the ELISA and the FTA-ABS test.^21^^,^^29^^,^^34^ In contrast, a study reported a Cohen’s kappa index between 0.31 and 0.73, depending on the performance of the different ELISA brands and the type of antibody detected (IgM or IgG).^30^ Finally, the FTA-ABS and VDRL tests also achieved an almost perfect agreement with results reported by Choi et al.^19^ (κ = 0.83) and Park et al.^18^ (κ = 0.85). It is important to note that despite the strong agreement among these tests, the diagnosis of syphilis should not rely on a single diagnostic test but rather on a combination of tests.

No cross-reactivity to other infectious diseases tested with the VDRL test was observed. On the other hand, we observed cross-reactivity when samples were tested with treponemal tests, especially with the FTA-ABS test. In fact, the positivity of the samples tested with the FTA-ABS test reached values of 14.7% for HCV, 12.5% for HIV, 8.8% for HBV, and 7.4% for HTLV, whereas the values for the ELISA were lower, i.e., 3.3% for HBV and 2.9% each for Chagas disease, HCV, and HTLV. In general, the number of samples testing positive in our study was low, which is consistent with a study that reported positive test results for HSV, Epstein-Barr virus, rheumatoid factor, heterophilic antibodies, and pregnancy.^38^ According to the manufacturer, the FTA-ABS test demonstrates a sensitivity and specificity of 99% and 95%, respectively. However, the manufacturer’s cross-reactivity analysis included only 27 samples positive for *Borrelia* and 27 samples from patients positive for *Treponema phagedenis*. This limited data set, as indicated in the diagnostic kit leaflet, may not be sufficient to determine the absence of cross-reactivity with other infectious-parasitic diseases due to the sample size and the restriction to two types of investigated diseases. Consequently, the results presented here suggest that the test can yield positive results for samples from patients with conditions other than syphilis, likely due to possible nonspecific reactions. Additionally, the FTA-ABS test exhibits lower sensitivity and specificity than other TTs,^12^ as it employs bacteria as an antigenic matrix and may occasionally produce false-positive (nonspecific fluorescence) and false-negative results.^41^ It is worth noting that using native proteins or crude extract in the ELISA rather than recombinant proteins is disadvantageous, as it may lead to reduced specificity and potentially increased cross-reactivity.^30^^,^^42^

No difference was found when assessing the sensitivity, specificity, and accuracy of the traditional and reverse algorithms. In contrast, Evren et al.^43^ found that the rate of missed diagnoses of the traditional algorithm was 42.5%. The reverse algorithm (99.85%) had a higher sensitivity than that of the traditional algorithm (57.49%). The false-positivity rate of the reverse algorithm was 0.02%. In fact, the discrepancy between the two algorithms was large, demonstrating the diagnostic performance of the reverse algorithm. A possible hypothesis for this finding is the large sample size used by the study authors (*n =* 4,789), which allowed for a more in-depth analysis. Furthermore, compared with this study, the difference in sensitivity between the treponemal tests may have been influenced by differences in the inherent characteristics of each diagnostic platform used (CMIA, ELISA, and FTA-ABS).

Buono et al.^35^ found that the reverse algorithm identified 38 additional seropositive individuals not detected by the traditional algorithm (+3.7% of positivity), and Rourk et al.^44^ showed that the reverse algorithm detected 21 patients with possible latent syphilis that were not detected by traditional algorithms. As a result, many clinical laboratories have adopted the reverse screening algorithm, in which samples are first tested with a TT and then screening-reactive samples are confirmed by an NTT. Currently, the CDC and the European Center for Disease Prevention and Control recommend the use of the reverse algorithm. It allows identification of patients with latent or early syphilis and increases the sensitivity of a serologic diagnosis of syphilis.

The major limitation of this study was the lack of syphilis samples from pregnant women and congenital syphilis for evaluating the diagnostic performance of all three commercial tests. Another limitation was the inability to obtain well-defined serologic samples for cross-reactivity evaluation, so only a small number of samples were analyzed in this study.

## CONCLUSION

We conclude that all tests were able to discriminate positive and negative samples, regardless of the diagnostic algorithm. Based on the performance parameters, we demonstrated the diagnostic suitability of the ELISA and VDRL test for the diagnosis of the different stages of syphilis (primary, latent, and secondary). It is also important to emphasize that the tests need to be improved in terms of eliminating false positives (cross-reactivity) with treponemal tests (ELISA and FTA-ABS) to achieve higher diagnostic accuracy.

## Supplemental Materials

10.4269/ajtmh.23-0238 Supplemental Materials
