# Supplementary material for: Performance Assessment of Treponemal and Nontreponemal Tests for the Diagnosis of Acquired Syphilis
Source: Am J Trop Med Hyg. 2024 Apr 9;110(6):1237–44. doi: 10.4269/ajtmh.23-0238 (PMC11154036; doi:10.4269/ajtmh.23-0238)
Supplement: Supplemental Materials [file tpmd230238.SD1.pdf]

Sample	Sera characterization		
	VDRL	ELISA (IgG)	FTA-ABS (IgG)
T. pallidum -negative sample 1	NON-REAGENT	0.30	NON-REAGENT
T. pallidum -negative sample 2	NON-REAGENT	0.32	NON-REAGENT
T. pallidum -negative sample 3	NON-REAGENT	0.21	NON-REAGENT
T. pallidum -negative sample 4	NON-REAGENT	0.29	NON-REAGENT
T. pallidum -negative sample 5	NON-REAGENT	0.28	NON-REAGENT
T. pallidum -negative sample 6	NON-REAGENT	0.24	NON-REAGENT
T. pallidum -negative sample 7	NON-REAGENT	0.27	NON-REAGENT
T. pallidum -negative sample 8	NON-REAGENT	0.26	NON-REAGENT
T. pallidum -negative sample 9	NON-REAGENT	0.24	NON-REAGENT
T. pallidum -negative sample 10	NON-REAGENT	0.24	NON-REAGENT
T. pallidum -negative sample 11	NON-REAGENT	0.21	NON-REAGENT
T. pallidum -negative sample 12	NON-REAGENT	0.24	NON-REAGENT
T. pallidum -negative sample 13	NON-REAGENT	0.44	NON-REAGENT
T. pallidum -negative sample 14	NON-REAGENT	0.25	NON-REAGENT
T. pallidum -negative sample 15	NON-REAGENT	0.25	NON-REAGENT
T. pallidum -negative sample 16	NON-REAGENT	0.25	NON-REAGENT
T. pallidum -negative sample 17	NON-REAGENT	0.28	NON-REAGENT
T. pallidum -negative sample 18	NON-REAGENT	0.22	NON-REAGENT
T. pallidum -negative sample 19	NON-REAGENT	0.25	NON-REAGENT
T. pallidum -negative sample 20	NON-REAGENT	0.25	NON-REAGENT
T. pallidum -negative sample 21	NON-REAGENT	0.36	NON-REAGENT
T. pallidum -negative sample 22	NON-REAGENT	0.49	NON-REAGENT
T. pallidum -negative sample 23	NON-REAGENT	0.24	NON-REAGENT
T. pallidum -negative sample 24	NON-REAGENT	0.29	NON-REAGENT
T. pallidum -negative sample 25	NON-REAGENT	0.23	NON-REAGENT
T. pallidum -negative sample 26	NON-REAGENT	0.29	NON-REAGENT
T. pallidum -negative sample 27	NON-REAGENT	0.27	NON-REAGENT
T. pallidum -negative sample 28	NON-REAGENT	0.24	NON-REAGENT
T. pallidum -negative sample 29	NON-REAGENT	0.26	NON-REAGENT
T. pallidum -negative sample 30	NON-REAGENT	0.35	NON-REAGENT
T. pallidum -negative sample 31	NON-REAGENT	0.28	NON-REAGENT
T. pallidum -negative sample 32	NON-REAGENT	0.34	NON-REAGENT
T. pallidum -negative sample 33	NON-REAGENT	0.30	NON-REAGENT
T. pallidum -negative sample 34	NON-REAGENT	0.26	NON-REAGENT
T. pallidum -negative sample 35	NON-REAGENT	0.23	NON-REAGENT
T. pallidum -negative sample 36	NON-REAGENT	0.23	NON-REAGENT
T. pallidum -negative sample 37	NON-REAGENT	0.27	NON-REAGENT
T. pallidum -negative sample 38	NON-REAGENT	0.25	NON-REAGENT
T. pallidum -negative sample 39	NON-REAGENT	0.33	NON-REAGENT
T. pallidum -negative sample 40	NON-REAGENT	0.25	NON-REAGENT
T. pallidum -negative sample 41	NON-REAGENT	0.27	NON-REAGENT
T. pallidum -negative sample 42	NON-REAGENT	0.27	NON-REAGENT
T. pallidum -negative sample 43	NON-REAGENT	0.20	NON-REAGENT

T. pallidum -negative sample 44	NON-REAGENT	0.28	NON-REAGENT
T. pallidum -negative sample 45	NON-REAGENT	0.27	NON-REAGENT
T. pallidum -negative sample 46	NON-REAGENT	0.38	NON-REAGENT
T. pallidum -negative sample 47	NON-REAGENT	0.26	NON-REAGENT
T. pallidum -negative sample 48	NON-REAGENT	0.24	NON-REAGENT
T. pallidum -negative sample 49	NON-REAGENT	0.24	NON-REAGENT
T. pallidum -negative sample 50	NON-REAGENT	0.27	NON-REAGENT
T. pallidum -negative sample 51	NON-REAGENT	0.33	NON-REAGENT
T. pallidum -negative sample 52	NON-REAGENT	0.30	NON-REAGENT
T. pallidum -negative sample 53	NON-REAGENT	0.32	NON-REAGENT
T. pallidum -negative sample 54	NON-REAGENT	0.31	NON-REAGENT
T. pallidum -negative sample 55	NON-REAGENT	0.33	NON-REAGENT
T. pallidum -negative sample 56	NON-REAGENT	0.25	NON-REAGENT
T. pallidum -negative sample 57	NON-REAGENT	0.27	NON-REAGENT
T. pallidum -negative sample 58	NON-REAGENT	0.23	NON-REAGENT
T. pallidum -negative sample 59	NON-REAGENT	0.24	NON-REAGENT
T. pallidum -negative sample 60	NON-REAGENT	0.23	NON-REAGENT
T. pallidum -negative sample 61	NON-REAGENT	0.25	NON-REAGENT
T. pallidum -negative sample 62	NON-REAGENT	0.27	NON-REAGENT
T. pallidum -negative sample 63	NON-REAGENT	0.25	NON-REAGENT
T. pallidum -negative sample 64	NON-REAGENT	0.29	NON-REAGENT
T. pallidum -negative sample 65	NON-REAGENT	0.32	NON-REAGENT
T. pallidum -negative sample 66	NON-REAGENT	0.60	NON-REAGENT
T. pallidum -negative sample 67	NON-REAGENT	0.20	NON-REAGENT
T. pallidum -negative sample 68	NON-REAGENT	0.32	NON-REAGENT
T. pallidum -negative sample 69	NON-REAGENT	0.30	NON-REAGENT
T. pallidum -negative sample 70	NON-REAGENT	0.27	NON-REAGENT
T. pallidum -negative sample 71	NON-REAGENT	0.24	NON-REAGENT
T. pallidum -negative sample 72	NON-REAGENT	0.24	NON-REAGENT
T. pallidum -negative sample 73	NON-REAGENT	0.24	NON-REAGENT
T. pallidum -negative sample 74	NON-REAGENT	0.32	NON-REAGENT
T. pallidum -negative sample 75	NON-REAGENT	0.30	NON-REAGENT
T. pallidum -negative sample 76	NON-REAGENT	0.27	NON-REAGENT
T. pallidum -negative sample 77	NON-REAGENT	0.27	NON-REAGENT
T. pallidum -negative sample 78	NON-REAGENT	0.32	NON-REAGENT
T. pallidum -negative sample 79	NON-REAGENT	0.30	NON-REAGENT
T. pallidum -negative sample 80	NON-REAGENT	0.31	NON-REAGENT
T. pallidum -negative sample 81	NON-REAGENT	0.24	NON-REAGENT
T. pallidum -negative sample 82	NON-REAGENT	0.29	NON-REAGENT
T. pallidum -negative sample 83	NON-REAGENT	0.24	NON-REAGENT
T. pallidum -negative sample 84	NON-REAGENT	0.27	NON-REAGENT
T. pallidum -negative sample 85	NON-REAGENT	0.25	NON-REAGENT
T. pallidum -negative sample 86	NON-REAGENT	0.24	NON-REAGENT
T. pallidum -negative sample 87	NON-REAGENT	0.31	NON-REAGENT
T. pallidum -negative sample 88	NON-REAGENT	0.25	NON-REAGENT
T. pallidum -negative sample 89	NON-REAGENT	0.33	NON-REAGENT

T. pallidum -negative sample 90	NON-REAGENT	0.41	NON-REAGENT
T. pallidum -negative sample 91	NON-REAGENT	0.29	NON-REAGENT
T. pallidum -negative sample 92	NON-REAGENT	0.25	NON-REAGENT
T. pallidum -negative sample 93	NON-REAGENT	0.26	NON-REAGENT
T. pallidum -negative sample 94	NON-REAGENT	0.33	NON-REAGENT
T. pallidum -negative sample 95	NON-REAGENT	0.35	NON-REAGENT
T. pallidum -negative sample 96	NON-REAGENT	0.25	NON-REAGENT
T. pallidum -negative sample 97	NON-REAGENT	0.26	NON-REAGENT
T. pallidum -negative sample 98	NON-REAGENT	0.24	NON-REAGENT
T. pallidum -negative sample 99	NON-REAGENT	0.28	NON-REAGENT
T. pallidum -negative sample 100	NON-REAGENT	0.21	NON-REAGENT
T. pallidum -negative sample 101	NON-REAGENT	0.26	NON-REAGENT
T. pallidum -negative sample 102	NON-REAGENT	0.26	NON-REAGENT
T. pallidum -negative sample 103	NON-REAGENT	0.31	NON-REAGENT
T. pallidum -negative sample 104	NON-REAGENT	0.31	NON-REAGENT
T. pallidum -negative sample 105	NON-REAGENT	0.23	NON-REAGENT
T. pallidum -negative sample 106	NON-REAGENT	0.28	NON-REAGENT
T. pallidum -negative sample 107	NON-REAGENT	0.24	NON-REAGENT
T. pallidum -negative sample 108	NON-REAGENT	0.29	NON-REAGENT
T. pallidum -negative sample 109	NON-REAGENT	0.21	NON-REAGENT
T. pallidum -negative sample 110	NON-REAGENT	0.24	NON-REAGENT
T. pallidum -negative sample 111	NON-REAGENT	0.34	NON-REAGENT
T. pallidum -negative sample 112	NON-REAGENT	0.26	NON-REAGENT
T. pallidum -negative sample 113	NON-REAGENT	0.49	NON-REAGENT
T. pallidum -negative sample 114	NON-REAGENT	0.30	NON-REAGENT
T. pallidum -negative sample 115	NON-REAGENT	0.29	NON-REAGENT
T. pallidum -negative sample 116	NON-REAGENT	0.25	NON-REAGENT
T. pallidum -negative sample 117	NON-REAGENT	0.25	NON-REAGENT
T. pallidum -negative sample 118	NON-REAGENT	0.84	NON-REAGENT
T. pallidum -negative sample 119	NON-REAGENT	0.25	NON-REAGENT
T. pallidum -negative sample 120	NON-REAGENT	0.23	NON-REAGENT
T. pallidum -negative sample 121	NON-REAGENT	0.21	NON-REAGENT
T. pallidum -negative sample 122	NON-REAGENT	0.23	NON-REAGENT
T. pallidum -negative sample 123	NON-REAGENT	0.23	NON-REAGENT
T. pallidum -negative sample 124	NON-REAGENT	0.26	NON-REAGENT
T. pallidum -negative sample 125	NON-REAGENT	0.31	NON-REAGENT
T. pallidum -negative sample 126	NON-REAGENT	0.23	NON-REAGENT
T. pallidum -negative sample 127	NON-REAGENT	0.23	NON-REAGENT
T. pallidum -negative sample 128	NON-REAGENT	0.23	NON-REAGENT
T. pallidum -negative sample 129	NON-REAGENT	0.33	NON-REAGENT
T. pallidum -negative sample 130	NON-REAGENT	0.36	NON-REAGENT
T. pallidum -negative sample 131	NON-REAGENT	0.34	NON-REAGENT
T. pallidum -negative sample 132	NON-REAGENT	0.28	NON-REAGENT
T. pallidum -negative sample 133	NON-REAGENT	0.29	NON-REAGENT
T. pallidum -negative sample 134	NON-REAGENT	0.48	NON-REAGENT
T. pallidum -negative sample 135	NON-REAGENT	0.33	NON-REAGENT

T. pallidum -negative sample 136	NON-REAGENT	0.40	NON-REAGENT
T. pallidum -negative sample 137	NON-REAGENT	0.32	NON-REAGENT
T. pallidum -negative sample 138	NON-REAGENT	0.27	NON-REAGENT
T. pallidum -negative sample 139	NON-REAGENT	0.30	NON-REAGENT
T. pallidum -negative sample 140	NON-REAGENT	0.24	NON-REAGENT
T. pallidum -negative sample 141	NON-REAGENT	0.27	NON-REAGENT
T. pallidum -negative sample 142	NON-REAGENT	0.25	NON-REAGENT
T. pallidum -negative sample 143	NON-REAGENT	0.40	NON-REAGENT
T. pallidum -negative sample 144	NON-REAGENT	0.29	NON-REAGENT
T. pallidum -negative sample 145	NON-REAGENT	0.25	NON-REAGENT
T. pallidum -negative sample 146	NON-REAGENT	0.29	NON-REAGENT
T. pallidum -negative sample 147	NON-REAGENT	0.48	NON-REAGENT
T. pallidum -negative sample 148	NON-REAGENT	0.33	NON-REAGENT
T. pallidum -negative sample 149	NON-REAGENT	0.25	NON-REAGENT
T. pallidum -negative sample 150	NON-REAGENT	0.35	NON-REAGENT
T. pallidum -negative sample 151	NON-REAGENT	0.30	NON-REAGENT
T. pallidum -negative sample 152	NON-REAGENT	0.27	NON-REAGENT
T. pallidum -negative sample 153	NON-REAGENT	0.25	NON-REAGENT
T. pallidum -negative sample 154	NON-REAGENT	0.27	NON-REAGENT
T. pallidum -negative sample 155	NON-REAGENT	0.45	NON-REAGENT
T. pallidum -negative sample 156	NON-REAGENT	0.44	NON-REAGENT
T. pallidum -negative sample 157	NON-REAGENT	0.24	NON-REAGENT
T. pallidum -negative sample 158	NON-REAGENT	0.24	NON-REAGENT
T. pallidum -negative sample 159	NON-REAGENT	0.36	NON-REAGENT
T. pallidum -negative sample 160	NON-REAGENT	0.31	NON-REAGENT
T. pallidum -negative sample 161	NON-REAGENT	0.30	NON-REAGENT
T. pallidum -negative sample 162	NON-REAGENT	0.31	NON-REAGENT
T. pallidum -negative sample 163	NON-REAGENT	0.26	NON-REAGENT
T. pallidum -negative sample 164	NON-REAGENT	0.25	NON-REAGENT
T. pallidum -negative sample 165	NON-REAGENT	0.26	NON-REAGENT
T. pallidum -negative sample 166	NON-REAGENT	0.36	NON-REAGENT
T. pallidum -negative sample 167	NON-REAGENT	0.24	NON-REAGENT
T. pallidum -negative sample 168	NON-REAGENT	0.26	NON-REAGENT
T. pallidum -negative sample 169	NON-REAGENT	0.29	NON-REAGENT
T. pallidum -negative sample 170	NON-REAGENT	0.27	NON-REAGENT
T. pallidum -negative sample 171	NON-REAGENT	0.23	NON-REAGENT
T. pallidum -negative sample 172	NON-REAGENT	0.25	NON-REAGENT
T. pallidum -negative sample 173	NON-REAGENT	0.25	NON-REAGENT
T. pallidum -negative sample 174	NON-REAGENT	0.31	NON-REAGENT
T. pallidum -negative sample 175	NON-REAGENT	0.26	NON-REAGENT
T. pallidum -negative sample 176	NON-REAGENT	0.31	NON-REAGENT
T. pallidum -negative sample 177	NON-REAGENT	0.36	NON-REAGENT
T. pallidum -negative sample 178	NON-REAGENT	0.46	NON-REAGENT
T. pallidum -negative sample 179	NON-REAGENT	0.34	NON-REAGENT
T. pallidum -negative sample 180	NON-REAGENT	0.77	NON-REAGENT
T. pallidum -negative sample 181	NON-REAGENT	0.48	NON-REAGENT

T. pallidum -negative sample 182	NON-REAGENT	0.34	NON-REAGENT
T. pallidum -negative sample 183	NON-REAGENT	0.36	NON-REAGENT
T. pallidum -negative sample 184	NON-REAGENT	0.38	NON-REAGENT
T. pallidum -negative sample 185	NON-REAGENT	0.43	NON-REAGENT
T. pallidum -negative sample 186	NON-REAGENT	0.34	NON-REAGENT
T. pallidum -negative sample 187	NON-REAGENT	0.35	NON-REAGENT
T. pallidum -negative sample 188	NON-REAGENT	0.29	NON-REAGENT
T. pallidum -negative sample 189	NON-REAGENT	0.66	NON-REAGENT
T. pallidum -negative sample 190	NON-REAGENT	0.98	NON-REAGENT
T. pallidum -negative sample 191	NON-REAGENT	0.41	NON-REAGENT
T. pallidum -negative sample 192	NON-REAGENT	0.36	REAGENT
T. pallidum-positive sample 1	1;1	8.92	REAGENT
T. pallidum-positive sample 2	1/2	11.07	REAGENT
T. pallidum-positive sample 3	1/16	8.10	REAGENT
T. pallidum-positive sample 4	1/32	11.31	REAGENT
T. pallidum-positive sample 5	1/16	10.80	REAGENT
T. pallidum-positive sample 6	1/128	7.22	REAGENT
T. pallidum-positive sample 7	1;1	3.94	REAGENT
T. pallidum-positive sample 8	1/64	10.24	REAGENT
T. pallidum-positive sample 9	1/256	11.09	REAGENT
T. pallidum-positive sample 10	1/64	8.91	REAGENT
T. pallidum-positive sample 11	1/4	9.25	REAGENT
T. pallidum-positive sample 12	1/8	7.50	REAGENT
T. pallidum-positive sample 13	1/1024	10.00	REAGENT
T. pallidum-positive sample 14	1/64	10.86	REAGENT
T. pallidum-positive sample 15	1/16	8.72	REAGENT
T. pallidum-positive sample 16	1/32	7.08	REAGENT
T. pallidum-positive sample 17	1/2	7.12	NON-REAGENT
T. pallidum-positive sample 18	1/8	2.31	NON-REAGENT
T. pallidum-positive sample 19	1/32	10.56	REAGENT
T. pallidum-positive sample 20	1/4	8.15	REAGENT
T. pallidum-positive sample 21	1/64	11.77	REAGENT
T. pallidum-positive sample 22	1/4	9.30	REAGENT
T. pallidum-positive sample 23	1/4	9.91	REAGENT
T. pallidum-positive sample 24	1/64	10.44	REAGENT
T. pallidum-positive sample 25	1/8	10.98	REAGENT
T. pallidum-positive sample 26	1/512	10.80	REAGENT
T. pallidum-positive sample 27	1/8	9.89	REAGENT
T. pallidum-positive sample 28	1/64	8.83	REAGENT
T. pallidum-positive sample 29	1/64	11.67	REAGENT
T. pallidum-positive sample 30	1/4	10.49	REAGENT
T. pallidum-positive sample 31	1/8	10.27	REAGENT
T. pallidum-positive sample 32	1/16	8.61	REAGENT
T. pallidum-positive sample 33	1/8	8.12	REAGENT
T. pallidum-positive sample 34	1/4	10.65	REAGENT
T. pallidum-positive sample 35	1/8	10.90	REAGENT

T. pallidum-positive sample 36	1/64	10.53	REAGENT
T. pallidum-positive sample 37	1/4	8.92	REAGENT
T. pallidum-positive sample 38	1/32	8.63	REAGENT
T. pallidum-positive sample 39	1/256	10.77	REAGENT
T. pallidum-positive sample 40	1/32	10.11	REAGENT
T. pallidum-positive sample 41	NON-REAGENT	6.62	NON-REAGENT
T. pallidum-positive sample 42	1/4	9.33	REAGENT
T. pallidum-positive sample 43	NON-REAGENT	10.56	REAGENT
T. pallidum-positive sample 44	1/64	9.29	REAGENT
T. pallidum-positive sample 45	1/16	9.80	REAGENT
T. pallidum-positive sample 46	1/4	9.82	REAGENT
T. pallidum-positive sample 47	1/8	8.06	REAGENT
T. pallidum-positive sample 48	1/512	5.50	REAGENT
T. pallidum-positive sample 49	1/512	11.44	REAGENT
T. pallidum-positive sample 50	1/128	6.86	REAGENT
T. pallidum-positive sample 51	1/512	8.47	REAGENT
T. pallidum-positive sample 52	1/8	9.20	REAGENT
T. pallidum-positive sample 53	1/256	10.57	REAGENT
T. pallidum-positive sample 54	1/4	9.35	REAGENT
T. pallidum-positive sample 55	1/64	7.75	REAGENT
T. pallidum-positive sample 56	1;1	9.04	REAGENT
T. pallidum-positive sample 57	1/64	6.15	REAGENT
T. pallidum-positive sample 58	1/8	10.35	REAGENT
T. pallidum-positive sample 59	1/4	3.77	REAGENT
T. pallidum-positive sample 60	1/2	4.14	REAGENT
T. pallidum-positive sample 61	1/4	8.56	REAGENT
T. pallidum-positive sample 62	1/4	7.25	REAGENT
T. pallidum-positive sample 63	1/16	10.80	REAGENT
T. pallidum-positive sample 64	1/8	10.23	REAGENT
T. pallidum-positive sample 65	1;1	8.46	REAGENT
T. pallidum-positive sample 66	1/64	7.04	REAGENT
T. pallidum-positive sample 67	1/16	9.30	REAGENT
T. pallidum-positive sample 68	1/16	11.46	REAGENT
T. pallidum-positive sample 69	1;1	7.08	REAGENT
T. pallidum-positive sample 70	1/16	7.59	REAGENT
T. pallidum-positive sample 71	1/8	10.54	REAGENT
T. pallidum-positive sample 72	1/128	9.20	REAGENT
T. pallidum-positive sample 73	NON-REAGENT	2.26	NON-REAGENT
T. pallidum-positive sample 74	1/4	1.04	NON-REAGENT
T. pallidum-positive sample 75	1/32	10.38	REAGENT
T. pallidum-positive sample 76	1/8	9.24	REAGENT
T. pallidum-positive sample 77	1/64	9.58	REAGENT
T. pallidum-positive sample 78	1/16	9.89	REAGENT
T. pallidum-positive sample 79	1/8	10.74	REAGENT
T. pallidum-positive sample 80	1/4	10.11	REAGENT
T. pallidum-positive sample 81	1/8	5.70	REAGENT

T. pallidum-positive sample 82	1/4	10.51	REAGENT
T. pallidum-positive sample 83	1/4	8.97	REAGENT
T. pallidum-positive sample 84	1/128	10.45	REAGENT
T. pallidum-positive sample 85	1/32	9.54	REAGENT
T. pallidum-positive sample 86	1/32	8.12	REAGENT
T. pallidum-positive sample 87	1/16	8.00	REAGENT
T. pallidum-positive sample 88	1/16	9.69	REAGENT
T. pallidum-positive sample 89	1/128	8.86	REAGENT
T. pallidum-positive sample 90	1/64	8.50	REAGENT
T. pallidum-positive sample 91	1/8	10.16	REAGENT
T. pallidum-positive sample 92	1/128	7.79	REAGENT
T. pallidum-positive sample 93	1/4	2.59	REAGENT
T. pallidum-positive sample 94	1/4	8.10	REAGENT
T. pallidum-positive sample 95	1/16	10.33	REAGENT
T. pallidum-positive sample 96	1/2	7.45	REAGENT
T. pallidum-positive sample 97	1/4	7.80	REAGENT
T. pallidum-positive sample 98	1/8	6.80	REAGENT
T. pallidum-positive sample 99	1/512	7.08	REAGENT
T. pallidum-positive sample 100	1/8	11.14	REAGENT
T. pallidum-positive sample 101	1/4	8.71	REAGENT
T. pallidum-positive sample 102	1/8	10.57	REAGENT
T. pallidum-positive sample 103	1/2	2.02	NON-REAGENT
T. pallidum-positive sample 104	1/32	10.60	REAGENT
T. pallidum-positive sample 105	1/16	10.90	REAGENT
T. pallidum-positive sample 106	1/16	11.22	REAGENT
T. pallidum-positive sample 107	1/128	8.10	REAGENT
T. pallidum-positive sample 108	1/8	7.45	REAGENT
T. pallidum-positive sample 109	1/128	11.80	REAGENT
T. pallidum-positive sample 110	1/2	10.68	REAGENT
T. pallidum-positive sample 111	1/4	9.95	REAGENT
T. pallidum-positive sample 112	1/32	11.50	REAGENT
T. pallidum-positive sample 113	1/32	11.52	REAGENT
T. pallidum-positive sample 114	1/128	8.59	REAGENT
T. pallidum-positive sample 115	1/16	10.49	REAGENT
T. pallidum-positive sample 116	1/64	7.16	REAGENT
T. pallidum-positive sample 117	1/4	10.75	REAGENT
T. pallidum-positive sample 118	1/256	9.19	REAGENT
T. pallidum-positive sample 119	1/8	10.40	REAGENT
T. pallidum-positive sample 120	1/256	11.45	REAGENT
T. pallidum-positive sample 121	1/4	10.45	REAGENT
T. pallidum-positive sample 122	1/128	11.07	REAGENT
T. pallidum-positive sample 123	1/128	10.92	REAGENT
T. pallidum-positive sample 124	1/512	11.23	REAGENT
T. pallidum-positive sample 125	1/4	8.21	REAGENT
T. pallidum-positive sample 126	1/16	11.35	REAGENT
T. pallidum-positive sample 127	1/64	11.45	REAGENT

T. pallidum-positive sample 128	1/128	11.13	REAGENT
T. pallidum-positive sample 129	1/16	8.57	REAGENT
T. pallidum-positive sample 130	1/1024	9.00	REAGENT
T. pallidum-positive sample 131	1/128	10.54	REAGENT
T. pallidum-positive sample 132	1/128	8.50	REAGENT
T. pallidum-positive sample 133	1/128	8.91	REAGENT
T. pallidum-positive sample 134	1/1024	8.11	REAGENT
T. pallidum-positive sample 135	1/64	9.85	REAGENT
T. pallidum-positive sample 136	1/256	8.92	REAGENT
T. pallidum-positive sample 137	1/2	9.25	NON-REAGENT
T. pallidum-positive sample 138	1/4	9.27	REAGENT
T. pallidum-positive sample 139	1/1024	9.57	REAGENT
T. pallidum-positive sample 140	1/2	9.74	REAGENT
T. pallidum-positive sample 141	1;1	9.29	REAGENT
T. pallidum-positive sample 142	1/2	6.67	REAGENT
T. pallidum-positive sample 143	1/128	11.03	REAGENT
T. pallidum-positive sample 144	1/128	6.68	REAGENT
T. pallidum-positive sample 145	1/512	9.16	REAGENT
T. pallidum-positive sample 146	1/32	10.37	REAGENT
T. pallidum-positive sample 147	1/32	11.06	REAGENT
T. pallidum-positive sample 148	1/128	10.45	REAGENT
T. pallidum-positive sample 149	1/32	9.59	REAGENT
T. pallidum-positive sample 150	1/16	7.03	REAGENT
T. pallidum-positive sample 151	1/4	7.96	REAGENT
T. pallidum-positive sample 152	1/32	8.86	REAGENT
T. pallidum-positive sample 153	1/32	7.00	REAGENT
T. pallidum-positive sample 154	1/256	6.68	REAGENT
T. pallidum-positive sample 155	1/2	8.06	REAGENT
T. pallidum-positive sample 156	1/128	8.09	REAGENT
T. pallidum-positive sample 157	1/8	9.06	REAGENT
T. pallidum-positive sample 158	1/1024	10.54	REAGENT
T. pallidum-positive sample 159	1/64	6.07	REAGENT
T. pallidum-positive sample 160	1/512	11.00	REAGENT
T. pallidum-positive sample 161	1/2	8.67	REAGENT
T. pallidum-positive sample 162	1/2	6.88	REAGENT
T. pallidum-positive sample 163	1/2	9.66	REAGENT
T. pallidum-positive sample 164	1/64	10.30	REAGENT
T. pallidum-positive sample 165	1;1	8.58	REAGENT
T. pallidum-positive sample 166	1/64	3.16	REAGENT
T. pallidum-positive sample 167	1/32	8.54	REAGENT
T. pallidum-positive sample 168	1/128	9.56	REAGENT
T. pallidum-positive sample 169	1/128	6.50	REAGENT
T. pallidum-positive sample 170	1/4	8.21	REAGENT
T. pallidum-positive sample 171	1;1	5.14	REAGENT
T. pallidum-positive sample 172	1/2	9.94	REAGENT
T. pallidum-positive sample 173	1/2	7.38	REAGENT

T. pallidum-positive sample 174	1/256	6.54	REAGENT
T. pallidum-positive sample 175	1/8	9.22	REAGENT
T. pallidum-positive sample 176	1/8	10.03	REAGENT
T. pallidum-positive sample 177	1/16	7.88	REAGENT
T. pallidum-positive sample 178	1/16	8.24	REAGENT
T. pallidum-positive sample 179	1/2	8.92	REAGENT
T. pallidum-positive sample 180	1/16	9.26	REAGENT
T. pallidum-positive sample 181	1/8	9.38	REAGENT
T. pallidum-positive sample 182	1/128	10.34	REAGENT
T. pallidum-positive sample 183	1/128	10.09	REAGENT
T. pallidum-positive sample 184	1/32	9.12	REAGENT
T. pallidum-positive sample 185	1/64	8.61	REAGENT
T. pallidum-positive sample 186	NON-REAGENT	9.94	REAGENT
T. pallidum-positive sample 187	1/8	10.36	REAGENT

Sample	Sera characterization		
	VDRL	ELISA (IgG)	FTA-ABS (IgG)
Chagas-positive sample 1	NON-REAGENT	0.32	NEGATIVE
Chagas-positive sample 2	NON-REAGENT	0.52	NEGATIVE
Chagas-positive sample 3	NON-REAGENT	0.28	NEGATIVE
Chagas-positive sample 4	NON-REAGENT	0.44	NEGATIVE
Chagas-positive sample 5	NON-REAGENT	0.48	NEGATIVE
Chagas-positive sample 6	NON-REAGENT	0.29	NEGATIVE
Chagas-positive sample 7	NON-REAGENT	0.30	NEGATIVE
Chagas-positive sample 8	NON-REAGENT	0.27	NEGATIVE
Chagas-positive sample 9	NON-REAGENT	0.30	NEGATIVE
Chagas-positive sample 10	NON-REAGENT	0.31	NEGATIVE
Chagas-positive sample 11	NON-REAGENT	0.27	NEGATIVE
Chagas-positive sample 12	NON-REAGENT	0.41	NEGATIVE
Chagas-positive sample 13	NON-REAGENT	0.38	NEGATIVE
Chagas-positive sample 14	NON-REAGENT	0.22	NEGATIVE
Chagas-positive sample 15	NON-REAGENT	0.48	NEGATIVE
Chagas-positive sample 16	NON-REAGENT	0.34	NEGATIVE
Chagas-positive sample 17	NON-REAGENT	0.32	NEGATIVE
Chagas-positive sample 18	NON-REAGENT	0.32	NEGATIVE
Chagas-positive sample 19	NON-REAGENT	0.38	NEGATIVE
Chagas-positive sample 20	NON-REAGENT	0.31	NEGATIVE
Chagas-positive sample 21	NON-REAGENT	0.39	NEGATIVE
Chagas-positive sample 22	NON-REAGENT	0.43	NEGATIVE
Chagas-positive sample 23	NON-REAGENT	0.25	NEGATIVE
Chagas-positive sample 24	NON-REAGENT	9.28	NEGATIVE
Chagas-positive sample 25	NON-REAGENT	0.32	NEGATIVE
Chagas-positive sample 26	NON-REAGENT	0.37	NEGATIVE
Chagas-positive sample 27	NON-REAGENT	0.31	NEGATIVE
Chagas-positive sample 28	NON-REAGENT	0.26	NEGATIVE
Chagas-positive sample 29	NON-REAGENT	0.36	NEGATIVE
Chagas-positive sample 30	NON-REAGENT	0.44	NEGATIVE
Chagas-positive sample 31	NON-REAGENT	0.60	NEGATIVE
Chagas-positive sample 32	NON-REAGENT	0.26	NEGATIVE
Chagas-positive sample 33	NON-REAGENT	0.25	NEGATIVE
Chagas-positive sample 34	NON-REAGENT	0.24	NEGATIVE
Chagas-positive sample 35	NON-REAGENT	0.32	NEGATIVE
Chagas-positive sample 36	NON-REAGENT	0.29	NEGATIVE
Chagas-positive sample 37	NON-REAGENT	0.30	NEGATIVE
Chagas-positive sample 38	NON-REAGENT	0.65	NEGATIVE
Chagas-positive sample 39	NON-REAGENT	0.26	NEGATIVE
Chagas-positive sample 40	NON-REAGENT	0.33	NEGATIVE
Chagas-positive sample 41	NON-REAGENT	1.15	NEGATIVE
Chagas-positive sample 42	NON-REAGENT	0.30	NEGATIVE
Chagas-positive sample 43	NON-REAGENT	0.29	NEGATIVE

Chagas-positive sample 44	NON-REAGENT	0.23	NEGATIVE
Chagas-positive sample 45	NON-REAGENT	0.23	NEGATIVE
Chagas-positive sample 46	NON-REAGENT	0.37	NEGATIVE
Chagas-positive sample 47	NON-REAGENT	0.28	NEGATIVE
Chagas-positive sample 48	NON-REAGENT	0.22	NEGATIVE
Chagas-positive sample 49	NON-REAGENT	0.35	NEGATIVE
Chagas-positive sample 50	NON-REAGENT	0.55	NEGATIVE
Chagas-positive sample 51	NON-REAGENT	0.25	NEGATIVE
Chagas-positive sample 52	NON-REAGENT	0.24	NEGATIVE
Chagas-positive sample 53	NON-REAGENT	0.37	NEGATIVE
Chagas-positive sample 54	NON-REAGENT	0.42	NEGATIVE
Chagas-positive sample 55	NON-REAGENT	0.29	NEGATIVE
Chagas-positive sample 56	NON-REAGENT	0.29	NEGATIVE
Chagas-positive sample 57	NON-REAGENT	0.29	NEGATIVE
Chagas-positive sample 58	NON-REAGENT	0.29	NEGATIVE
Chagas-positive sample 59	NON-REAGENT	0.30	NEGATIVE
Chagas-positive sample 60	NON-REAGENT	0.32	NEGATIVE
Chagas-positive sample 61	NON-REAGENT	0.18	NEGATIVE
Chagas-positive sample 62	NON-REAGENT	0.27	NEGATIVE
Chagas-positive sample 63	NON-REAGENT	0.27	NEGATIVE
Chagas-positive sample 64	NON-REAGENT	0.28	NEGATIVE
Chagas-positive sample 65	NON-REAGENT	0.27	NEGATIVE
Chagas-positive sample 66	NON-REAGENT	0.43	NEGATIVE
Chagas-positive sample 67	NON-REAGENT	0.21	NEGATIVE
Chagas-positive sample 68	NON-REAGENT	0.24	NEGATIVE
HBV-positive sample 1	NON-REAGENT	0.36	NEGATIVE
HBV-positive sample 2	NON-REAGENT	0.41	NEGATIVE
HBV-positive sample 3	NON-REAGENT	0.33	NEGATIVE
HBV-positive sample 4	NON-REAGENT	0.35	NEGATIVE
HBV-positive sample 5	NON-REAGENT	0.35	NEGATIVE
HBV-positive sample 6	NON-REAGENT	0.48	NEGATIVE
HBV-positive sample 7	NON-REAGENT	0.42	NEGATIVE
HBV-positive sample 8	NON-REAGENT	0.30	NEGATIVE
HBV-positive sample 9	NON-REAGENT	0.67	NEGATIVE
HBV-positive sample 10	NON-REAGENT	0.57	NEGATIVE
HBV-positive sample 11	NON-REAGENT	0.68	NEGATIVE
HBV-positive sample 12	NON-REAGENT	0.40	NEGATIVE
HBV-positive sample 13	NON-REAGENT	0.55	NEGATIVE
HBV-positive sample 14	NON-REAGENT	0.62	NEGATIVE
HBV-positive sample 15	NON-REAGENT	0.37	NEGATIVE
HBV-positive sample 16	NON-REAGENT	0.35	NEGATIVE
HBV-positive sample 17	NON-REAGENT	0.41	NEGATIVE
HBV-positive sample 18	NON-REAGENT	0.37	NEGATIVE
HBV-positive sample 19	NON-REAGENT	0.32	NEGATIVE
HBV-positive sample 20	NON-REAGENT	0.31	NEGATIVE
HBV-positive sample 21	NON-REAGENT	0.24	NEGATIVE

HBV-positive sample 22	NON-REAGENT	0.35	NEGATIVE
HBV-positive sample 23	NON-REAGENT	0.51	NEGATIVE
HBV-positive sample 24	NON-REAGENT	0.41	NEGATIVE
HBV-positive sample 25	NON-REAGENT	0.90	NEGATIVE
HBV-positive sample 26	NON-REAGENT	0.53	NEGATIVE
HBV-positive sample 27	NON-REAGENT	0.51	NEGATIVE
HBV-positive sample 28	NON-REAGENT	0.36	NEGATIVE
HBV-positive sample 29	NON-REAGENT	0.36	NEGATIVE
HBV-positive sample 30	NON-REAGENT	0.48	NEGATIVE
HBV-positive sample 31	NON-REAGENT	0.46	NEGATIVE
HBV-positive sample 32	NON-REAGENT	0.37	NEGATIVE
HBV-positive sample 33	NON-REAGENT	0.60	NEGATIVE
HBV-positive sample 34	NON-REAGENT	0.45	NEGATIVE
HBV-positive sample 35	NON-REAGENT	1.32	NEGATIVE
HBV-positive sample 36	NON-REAGENT	0.34	NEGATIVE
HBV-positive sample 37	NON-REAGENT	0.27	NEGATIVE
HBV-positive sample 38	NON-REAGENT	0.45	NEGATIVE
HBV-positive sample 39	NON-REAGENT	0.34	NEGATIVE
HBV-positive sample 40	NON-REAGENT	0.39	NEGATIVE
HBV-positive sample 41	NON-REAGENT	0.29	NEGATIVE
HBV-positive sample 42	NON-REAGENT	0.36	NEGATIVE
HBV-positive sample 43	NON-REAGENT	0.70	NEGATIVE
HBV-positive sample 44	NON-REAGENT	0.42	NEGATIVE
HBV-positive sample 45	NON-REAGENT	0.37	NEGATIVE
HBV-positive sample 46	NON-REAGENT	0.28	NEGATIVE
HBV-positive sample 47	NON-REAGENT	0.40	NEGATIVE
HBV-positive sample 48	NON-REAGENT	0.47	NEGATIVE
HBV-positive sample 49	NON-REAGENT	0.24	NEGATIVE
HBV-positive sample 50	NON-REAGENT	0.32	NEGATIVE
HBV-positive sample 51	NON-REAGENT	0.34	POSITIVE
HBV-positive sample 52	NON-REAGENT	0.29	NEGATIVE
HBV-positive sample 53	NON-REAGENT	0.29	POSITIVE
HBV-positive sample 54	NON-REAGENT	0.26	POSITIVE
HBV-positive sample 55	NON-REAGENT	0.36	NEGATIVE
HBV-positive sample 56	NON-REAGENT	0.28	NEGATIVE
HBV-positive sample 57	NON-REAGENT	0.27	NEGATIVE
HBV-positive sample 58	NON-REAGENT	0.47	NEGATIVE
HBV-positive sample 59	NON-REAGENT	0.37	NEGATIVE
HBV-positive sample 60	NON-REAGENT	0.20	POSITIVE
HBV-positive sample 61	NON-REAGENT	1.68	NEGATIVE
HBV-positive sample 62	NON-REAGENT	0.36	NEGATIVE
HBV-positive sample 63	NON-REAGENT	0.25	NEGATIVE
HBV-positive sample 64	NON-REAGENT	0.31	NEGATIVE
HBV-positive sample 65	NON-REAGENT	2.73	NEGATIVE
HBV-positive sample 66	NON-REAGENT	0.39	POSITIVE
HBV-positive sample 67	NON-REAGENT	0.40	NEGATIVE

HBV-positive sample 68	NON-REAGENT	0.34	NEGATIVE
HBV-positive sample 69	NON-REAGENT	0.38	NEGATIVE
HBV-positive sample 70	NON-REAGENT	0.35	NEGATIVE
HBV-positive sample 71	NON-REAGENT	0.28	NEGATIVE
HBV-positive sample 72	NON-REAGENT	0.35	NEGATIVE
HBV-positive sample 73	NON-REAGENT	0.24	NEGATIVE
HBV-positive sample 74	NON-REAGENT	0.26	POSITIVE
HBV-positive sample 75	NON-REAGENT	0.27	NEGATIVE
HBV-positive sample 76	NON-REAGENT	0.29	NEGATIVE
HBV-positive sample 77	NON-REAGENT	0.24	NEGATIVE
HBV-positive sample 78	NON-REAGENT	0.35	NEGATIVE
HBV-positive sample 79	NON-REAGENT	0.35	NEGATIVE
HBV-positive sample 80	NON-REAGENT	0.28	NEGATIVE
HBV-positive sample 81	NON-REAGENT	0.35	NEGATIVE
HBV-positive sample 82	NON-REAGENT	0.28	NEGATIVE
HBV-positive sample 83	NON-REAGENT	0.21	NEGATIVE
HBV-positive sample 84	NON-REAGENT	0.29	NEGATIVE
HBV-positive sample 85	NON-REAGENT	0.26	NEGATIVE
HBV-positive sample 86	NON-REAGENT	0.24	POSITIVE
HBV-positive sample 87	NON-REAGENT	0.29	NEGATIVE
HBV-positive sample 88	NON-REAGENT	0.26	NEGATIVE
HBV-positive sample 89	NON-REAGENT	0.29	POSITIVE
HBV-positive sample 90	NON-REAGENT	0.34	NEGATIVE
HBV-positive sample 91	NON-REAGENT	0.34	NEGATIVE
HCV-positive sample 1	NON-REAGENT	0.44	NEGATIVE
HCV-positive sample 2	NON-REAGENT	0.39	POSITIVE
HCV-positive sample 3	NON-REAGENT	0.74	NEGATIVE
HCV-positive sample 4	NON-REAGENT	0.34	NEGATIVE
HCV-positive sample 5	NON-REAGENT	0.48	NEGATIVE
HCV-positive sample 6	NON-REAGENT	0.69	NEGATIVE
HCV-positive sample 7	NON-REAGENT	0.40	NEGATIVE
HCV-positive sample 8	NON-REAGENT	0.53	NEGATIVE
HCV-positive sample 9	NON-REAGENT	0.39	NEGATIVE
HCV-positive sample 10	NON-REAGENT	0.33	NEGATIVE
HCV-positive sample 11	NON-REAGENT	0.47	NEGATIVE
HCV-positive sample 12	NON-REAGENT	0.59	POSITIVE
HCV-positive sample 13	NON-REAGENT	0.49	NEGATIVE
HCV-positive sample 14	NON-REAGENT	0.43	POSITIVE
HCV-positive sample 15	NON-REAGENT	0.32	NEGATIVE
HCV-positive sample 16	NON-REAGENT	0.63	NEGATIVE
HCV-positive sample 17	NON-REAGENT	0.59	NEGATIVE
HCV-positive sample 18	NON-REAGENT	0.31	POSITIVE
HCV-positive sample 19	NON-REAGENT	0.52	NEGATIVE
HCV-positive sample 20	NON-REAGENT	0.64	POSITIVE
HCV-positive sample 21	NON-REAGENT	0.61	NEGATIVE
HCV-positive sample 22	NON-REAGENT	0.74	NEGATIVE

HCV-positive sample 23	NON-REAGENT	0.38	NEGATIVE
HCV-positive sample 24	NON-REAGENT	0.54	NEGATIVE
HCV-positive sample 25	NON-REAGENT	0.37	NEGATIVE
HCV-positive sample 26	NON-REAGENT	0.47	NEGATIVE
HCV-positive sample 27	NON-REAGENT	0.41	NEGATIVE
HCV-positive sample 28	NON-REAGENT	0.37	POSITIVE
HCV-positive sample 29	NON-REAGENT	0.43	NEGATIVE
HCV-positive sample 30	NON-REAGENT	0.33	NEGATIVE
HCV-positive sample 31	NON-REAGENT	0.21	NEGATIVE
HCV-positive sample 32	NON-REAGENT	0.44	NEGATIVE
HCV-positive sample 33	NON-REAGENT	0.19	POSITIVE
HCV-positive sample 34	NON-REAGENT	0.39	NEGATIVE
HCV-positive sample 35	NON-REAGENT	0.39	NEGATIVE
HCV-positive sample 36	NON-REAGENT	0.28	NEGATIVE
HCV-positive sample 37	NON-REAGENT	0.23	NEGATIVE
HCV-positive sample 38	NON-REAGENT	0.39	NEGATIVE
HCV-positive sample 39	NON-REAGENT	0.19	NEGATIVE
HCV-positive sample 40	NON-REAGENT	0.48	NEGATIVE
HCV-positive sample 41	NON-REAGENT	0.22	NEGATIVE
HCV-positive sample 42	NON-REAGENT	0.33	POSITIVE
HCV-positive sample 43	NON-REAGENT	0.27	POSITIVE
HCV-positive sample 44	NON-REAGENT	0.29	NEGATIVE
HCV-positive sample 45	NON-REAGENT	0.34	NEGATIVE
HCV-positive sample 46	NON-REAGENT	0.27	NEGATIVE
HCV-positive sample 47	NON-REAGENT	0.26	NEGATIVE
HCV-positive sample 48	NON-REAGENT	0.67	NEGATIVE
HCV-positive sample 49	NON-REAGENT	9.99	NEGATIVE
HCV-positive sample 50	NON-REAGENT	0.25	NEGATIVE
HCV-positive sample 51	NON-REAGENT	0.24	NEGATIVE
HCV-positive sample 52	NON-REAGENT	0.30	NEGATIVE
HCV-positive sample 53	NON-REAGENT	0.22	NEGATIVE
HCV-positive sample 54	NON-REAGENT	0.22	NEGATIVE
HCV-positive sample 55	NON-REAGENT	9.90	POSITIVE
HCV-positive sample 56	NON-REAGENT	0.29	NEGATIVE
HCV-positive sample 57	NON-REAGENT	0.34	NEGATIVE
HCV-positive sample 58	NON-REAGENT	0.32	NEGATIVE
HCV-positive sample 59	NON-REAGENT	0.42	NEGATIVE
HCV-positive sample 60	NON-REAGENT	0.29	NEGATIVE
HCV-positive sample 61	NON-REAGENT	0.31	NEGATIVE
HCV-positive sample 62	NON-REAGENT	0.25	NEGATIVE
HCV-positive sample 63	NON-REAGENT	0.25	NEGATIVE
HCV-positive sample 64	NON-REAGENT	0.25	NEGATIVE
HCV-positive sample 65	NON-REAGENT	0.30	NEGATIVE
HCV-positive sample 66	NON-REAGENT	0.28	NEGATIVE
HCV-positive sample 67	NON-REAGENT	0.24	NEGATIVE
HCV-positive sample 68	NON-REAGENT	0.26	NEGATIVE

HCV-positive sample 69	NON-REAGENT	0.24	NEGATIVE
HCV-positive sample 70	NON-REAGENT	0.31	NEGATIVE
HCV-positive sample 71	NON-REAGENT	0.32	NEGATIVE
HCV-positive sample 72	NON-REAGENT	0.29	NEGATIVE
HIV-positive sample 1	NON-REAGENT	0.28	NEGATIVE
HIV-positive sample 2	NON-REAGENT	0.42	NEGATIVE
HIV-positive sample 3	NON-REAGENT	0.26	NEGATIVE
HIV-positive sample 4	NON-REAGENT	0.24	POSITIVE
HIV-positive sample 5	NON-REAGENT	0.27	NEGATIVE
HIV-positive sample 6	NON-REAGENT	0.31	NEGATIVE
HIV-positive sample 7	NON-REAGENT	0.30	NEGATIVE
HIV-positive sample 8	NON-REAGENT	0.24	NEGATIVE
HIV-positive sample 9	NON-REAGENT	0.28	POSITIVE
HIV-positive sample 10	NON-REAGENT	0.22	NEGATIVE
HIV-positive sample 11	NON-REAGENT	0.27	POSITIVE
HIV-positive sample 12	NON-REAGENT	0.23	NEGATIVE
HIV-positive sample 13	NON-REAGENT	0.79	NEGATIVE
HIV-positive sample 14	NON-REAGENT	0.33	NEGATIVE
HIV-positive sample 15	NON-REAGENT	0.25	NEGATIVE
HIV-positive sample 16	NON-REAGENT	0.32	NEGATIVE
HIV-positive sample 17	NON-REAGENT	0.26	NEGATIVE
HIV-positive sample 18	NON-REAGENT	0.44	NEGATIVE
HIV-positive sample 19	NON-REAGENT	0.25	NEGATIVE
HIV-positive sample 20	NON-REAGENT	0.24	NEGATIVE
HIV-positive sample 21	NON-REAGENT	0.37	NEGATIVE
HIV-positive sample 22	NON-REAGENT	0.30	NEGATIVE
HIV-positive sample 23	NON-REAGENT	0.25	NEGATIVE
HIV-positive sample 24	NON-REAGENT	0.33	NEGATIVE
HTLV-positive sample 1	NON-REAGENT	0.27	POSITIVE
HTLV-positive sample 2	NON-REAGENT	0.28	NEGATIVE
HTLV-positive sample 3	NON-REAGENT	0.22	NEGATIVE
HTLV-positive sample 4	NON-REAGENT	0.26	NEGATIVE
HTLV-positive sample 5	NON-REAGENT	0.27	NEGATIVE
HTLV-positive sample 6	NON-REAGENT	0.27	NEGATIVE
HTLV-positive sample 7	NON-REAGENT	0.25	NEGATIVE
HTLV-positive sample 8	NON-REAGENT	0.72	NEGATIVE
HTLV-positive sample 9	NON-REAGENT	0.21	NEGATIVE
HTLV-positive sample 10	NON-REAGENT	0.24	POSITIVE
HTLV-positive sample 11	NON-REAGENT	0.28	NEGATIVE
HTLV-positive sample 12	NON-REAGENT	0.28	NEGATIVE
HTLV-positive sample 13	NON-REAGENT	0.39	NEGATIVE
HTLV-positive sample 14	NON-REAGENT	0.36	NEGATIVE
HTLV-positive sample 15	NON-REAGENT	0.50	NEGATIVE
HTLV-positive sample 16	NON-REAGENT	0.27	NEGATIVE
HTLV-positive sample 17	NON-REAGENT	0.23	POSITIVE
HTLV-positive sample 18	NON-REAGENT	0.28	NEGATIVE

HTLV-positive sample 19	NON-REAGENT	0.39	NEGATIVE
HTLV-positive sample 20	NON-REAGENT	0.27	NEGATIVE
HTLV-positive sample 21	NON-REAGENT	0.25	NEGATIVE
HTLV-positive sample 22	NON-REAGENT	8.72	NEGATIVE
HTLV-positive sample 23	NON-REAGENT	0.25	NEGATIVE
HTLV-positive sample 24	NON-REAGENT	0.28	NEGATIVE
HTLV-positive sample 25	NON-REAGENT	0.26	NEGATIVE
HTLV-positive sample 26	NON-REAGENT	0.25	NEGATIVE
HTLV-positive sample 27	NON-REAGENT	0.33	NEGATIVE
HTLV-positive sample 28	NON-REAGENT	0.32	NEGATIVE
HTLV-positive sample 29	NON-REAGENT	0.32	NEGATIVE
HTLV-positive sample 30	NON-REAGENT	0.26	NEGATIVE
HTLV-positive sample 31	NON-REAGENT	0.26	NEGATIVE
HTLV-positive sample 32	NON-REAGENT	0.25	NEGATIVE
HTLV-positive sample 33	NON-REAGENT	0.27	NEGATIVE
HTLV-positive sample 34	NON-REAGENT	0.43	NEGATIVE
HTLV-positive sample 35	NON-REAGENT	0.36	NEGATIVE
HTLV-positive sample 36	NON-REAGENT	0.35	NEGATIVE
HTLV-positive sample 37	NON-REAGENT	0.32	NEGATIVE
HTLV-positive sample 38	NON-REAGENT	0.25	NEGATIVE
HTLV-positive sample 39	NON-REAGENT	0.24	NEGATIVE
HTLV-positive sample 40	NON-REAGENT	0.29	NEGATIVE
HTLV-positive sample 41	NON-REAGENT	0.27	NEGATIVE
HTLV-positive sample 42	NON-REAGENT	0.26	NEGATIVE
HTLV-positive sample 43	NON-REAGENT	0.26	NEGATIVE
HTLV-positive sample 44	NON-REAGENT	0.32	NEGATIVE
HTLV-positive sample 45	NON-REAGENT	0.24	NEGATIVE
HTLV-positive sample 46	NON-REAGENT	0.30	NEGATIVE
HTLV-positive sample 47	NON-REAGENT	0.25	NEGATIVE
HTLV-positive sample 48	NON-REAGENT	0.25	NEGATIVE
HTLV-positive sample 49	NON-REAGENT	0.29	NEGATIVE
HTLV-positive sample 50	NON-REAGENT	0.26	NEGATIVE
HTLV-positive sample 51	NON-REAGENT	0.31	NEGATIVE
HTLV-positive sample 52	NON-REAGENT	0.33	NEGATIVE
HTLV-positive sample 53	NON-REAGENT	0.29	NEGATIVE
HTLV-positive sample 54	NON-REAGENT	0.31	NEGATIVE
HTLV-positive sample 55	NON-REAGENT	0.38	NEGATIVE
HTLV-positive sample 56	NON-REAGENT	0.30	NEGATIVE
HTLV-positive sample 57	NON-REAGENT	0.26	NEGATIVE
HTLV-positive sample 58	NON-REAGENT	0.35	NEGATIVE
HTLV-positive sample 59	NON-REAGENT	0.27	NEGATIVE
HTLV-positive sample 60	NON-REAGENT	0.25	NEGATIVE
HTLV-positive sample 61	NON-REAGENT	0.24	POSITIVE
HTLV-positive sample 62	NON-REAGENT	0.27	NEGATIVE
HTLV-positive sample 63	NON-REAGENT	0.27	NEGATIVE
HTLV-positive sample 64	NON-REAGENT	0.32	NEGATIVE

HTLV-positive sample 65	NON-REAGENT	0.31	POSITIVE
HTLV-positive sample 66	NON-REAGENT	0.44	NEGATIVE
HTLV-positive sample 67	NON-REAGENT	0.33	NEGATIVE
HTLV-positive sample 68	NON-REAGENT	2.97	NEGATIVE
